# Effect of Postnatal Myostatin Inhibition on Bite Mechanics in Mice

**DOI:** 10.1371/journal.pone.0134854

**Published:** 2015-08-07

**Authors:** Susan H. Williams, Nicholas R. Lozier, Stéphane J. Montuelle, Sonsoles de Lacalle

**Affiliations:** Department of Biomedical Sciences, Ohio University Heritage College of Osteopathic Medicine, Athens, Ohio, United States of America; Institut de Myologie, FRANCE

## Abstract

As a negative regulator of muscle size, myostatin (Mstn) impacts the force-production capabilities of skeletal muscles. In the masticatory system, measures of temporalis-stimulated bite forces in constitutive myostatin KOs suggest an absolute, but not relative, increase in jaw-muscle force. Here, we assess the phenotypic and physiologic impact of postnatal myostatin inhibition on bite mechanics using an inducible conditional KO mouse in which myostatin is inhibited with doxycycline (DOX). Given the increased control over the timing of gene inactivation in this model, it may be more clinically-relevant for developing interventions for age-associated changes in the musculoskeletal system. DOX was administered for 12 weeks starting at age 4 months, during which time food intake was monitored. Sex, age and strain-matched controls were given the same food without DOX. Bite forces were recorded just prior to euthanasia after which muscle and skeletal data were collected. Food intake did not differ between control or DOX animals within each sex. DOX males were significantly larger and had significantly larger masseters than controls, but DOX and control females did not differ. Although there was a tendency towards higher absolute bite forces in DOX animals, this was not significant, and bite forces normalized to masseter mass did not differ. Mechanical advantage for incisor biting increased in the DOX group due to longer masseter moment arms, likely due to a more anteriorly-placed masseter insertion. Despite only a moderate increase in bite force in DOX males and none in DOX females, the increase in masseter mass in males indicates a potentially positive impact on jaw muscles. Our data suggest a sexual dimorphism in the role of mstn, and as such investigations into the sex-specific outcomes is warranted.

## Introduction

The protein myostatin (Mstn) is a member of the TGF-8 beta family that negatively regulates muscle size. Loss or mutations in the myostatin gene result in muscular enlargement due primarily to muscle cell hyperplasia [1, 2]. Indeed, in homozygous myostatin knockout (KO, or *Mstn* ^-/-^) mice, muscle mass can be almost twice that of controls (e.g., [2–6]). Whether muscular hypertrophy in the constitutive knockout is associated with an overall increase in force production capabilities is debated, but significant evidence demonstrates that complete absence of myostatin may not alter the absolute force-generating capacity in some muscles, and in others, it may result in a decrease in force relative to muscle size [4–6].

While the vast majority of our understanding of Mstn inhibition comes from studies of post-cranial muscles, we know that cranial muscles are also significantly affected. The constitutive *Mstn* ^-/-^ mouse model has significantly larger temporalis, masseter, and medial and lateral pterygoid muscles than wild-type controls. Moreover, stimulation of the temporalis to tetanus in anesthetized *Mstn* KOs results in significantly larger absolute (but not relative) bite forces compared to controls [7–10]. An increase in temporalis size in Mstn-deficient mice is also accompanied by an increase in the proportion of type II myofibers and, contrary to limb muscles, show a decrease in type II fiber diameter [7]. These data, coupled with morphologic findings for the skull in *Mstn* ^-/-^ mice (e.g., shorter crania, longer and rounder mandibles), provide further evidence of an altered craniofacial loading environment due to Mstn deficiency [8–14]. Endocranial volume and brain size are also significantly reduced in *Mstn* ^-/-^ mice, which has been interpreted as a potential influence of masticatory muscle hypertrophy on brain growth [10].

Despite the usefulness of constitutive KOs for understanding the role of Mstn in regulating skeletal muscle form and function, gene inactivation occurs at the embryonic stage, and this can impact postnatal growth and development. In the craniofacial skeleton of *Mstn* ^-/-^, there appear to be early growth deficiencies coupled with compensatory changes in the skull associated with increased muscle mass [9, 14]. Thus, this model may have limited applicability for developing treatments for pathologic states in which changes in skeletal muscles occur after musculoskeletal or biological maturity, such as age-related loss of muscle strength (dynapenia) or mass (sarcopenia), both of which can affect oral function [15]. In contrast, inducible conditional KOs where select genes may be silenced experimentally have become increasingly popular given the control over the timing of postnatal gene inactivation.

The aim of this study is to determine the effects of postnatal disruption of *Mstn* gene function on jaw-muscle anatomy and performance using an inducible KO mouse model in which the *Mstn* gene is inactivated near the time of skeletal maturity. In this model, a critical segment of the *Mstn* gene (exon 3) is flanked by loxP (floxed) and also contains an inducible Cre recombinase transgene. Treatment with doxycycline (DOX) renders the *Mstn* gene non-functional by excising the DNA flanked by the LoxP sequences. The power of this model is the ability to turn the *Mstn* gene 'off' at any time in the adult, thus avoiding interference with embryonic or postnatal development. We compare body mass, masseter muscle weight and voluntary incisor bite forces in this conditional KO with and without (control) administration of DOX. Given recent evidence that the postnatal administration of Mstn inhibitors alters the phenotype and force generating properties of postcranial muscles (e.g., [16–19]), we expect differences between DOX-treated animals and controls in body size, muscle size and maximum bite force. In order to rule out any potential biomechanical effects on bite force of altered masticatory configuration, we also compare the mechanical advantage for incisor biting, an estimate of the moment arm of the superficial masseter and the load arm associated with incisor biting between the groups. Mechanical advantage improves through anterior shifts in muscle attachment, posterior repositioning of the incisor bite point, or both. In order to fully understand the behavioral context in which any differences in performance and morphology occur, we also evaluate weekly food consumption for DOX and control animals.

## Materials and Methods

### Animal Model

We use a mouse model in which a critical segment of the *Mstn* gene (exon 3) is flanked by loxP (floxed) and also contains a Dox-inducible Cre recombinase transgene. Treatment with Dox renders the *Mstn* gene non-functional (by excising the DNA flanked by the LoxP sequences) and results in a reduction of Mstn mRNA levels. Our experimental animal is the result of breeding the B6;129S7-Mstn^tm1Swe^l/J mouse strain (Jackson Laboratories stock number 012685) and the HSA-rtTA/TRE-Cre mutant mouse strain (Jackson Laboratories stock number 012433). The B6;129S7-Mstn^tm1Swe^l/J strain is a targeted mutation strain that has Exon 3 of the *Mstn* gene flanked by loxP sites. When crossed with a Cre recombinase-expressing strain, tissue-specific expression of the gene occurs. HSA-rtTA/TRE-Cre mutant mouse strain has a tetracycline (doxycycline) inducible Cre-mediated recombination system specific for skeletal muscle. Further details on the development of these strains is provided by Jackson Laboratories. A similar animal model, using a tamoxifen-inducible transgene, has demonstrated that following 5 days of treatment with tamoxifen, >99% of Mstn mRNA is lost in the gastrocnemius muscle and the gastrocnemius and quadriceps masses increase by 25% [18–20]. The increase in size is due to fiber hypertrophy and to the expansion of the myonuclear domain. Here, we use the doxycycline-inducible construct because of the secondary effects of tamoxifen in mice that could potentially impact the study [21, 22].

This study was carried out in accordance with the recommendations in the Guide for the Care and Use of Laboratory Animals of the National Institutes of Health. The protocol was approved by the Ohio University Institutional Animal Care and Use Committee (Protocol Number: 13-H-016). No adverse events occurred or experimental modifications were made during the course of this study.

### Husbandry and DOX administration

Animals were obtained after weaning and were housed in groups of 4, separated by sex. They were kept on a 12:12 light:dark cycle, with food and water ad libitum, using a standard diet (RMH-3000 chow). At age 4 months, half of each sex was started on a special diet (Harlan's Teklad 8640) containing 200 mg/kg of DOX. Final sample sizes for each group are as follows: male control = 8; male DOX = 10; female control = 10; female DOX = 10. Teklad 8640 and RMH3000 have identical protein and fat levels (22% protein, ~5% fat) and contain similar ingredients (wheat, soybean meal, corn, wheat middlings, and fish meal). Food was weighed at initial provisioning and weekly thereafter before supplementing with a known amount. A weekly estimate of food consumed per animal was calculated as the total weight of food consumed each week divided by the number of animals in the cage.

### Bite Force Measurements

Natural (unstimulated) bite force was measured using a bite force transducer based on the design of Dechow and Carlson [23] with modifications and recording techniques described in Williams et al. [24]. Prior to each session, the transducer was calibrated with 100–1000 g calibration weights. Each calibration procedure showed a highly correlated (R^2^>0.98) linear relationship between voltage output and weight.

Bite forces were measured in the DOX-treated mice after 12 weeks of DOX treatment. Bite forces in the age and sex-matched controls were tested at the same time. Mice were scruffed and the transducer was presented directly in front of their mouth until they willingly bit the transducer at the incisors. Each animal participated in three recording sessions on three separate days, and all data were recorded for each individual within the same week. Each session produced multiple bites. The linear relationship resulting from each calibration procedure was used to convert voltage output from each bite within that recording session to kg and subsequently Newtons. For each animal, the average maximum bite force is based on the largest bite from each recording session. All statistical analyses are based on this average.

### Dissections and phenotypic measurements

After bite forces were recorded, all animals were weighed to the nearest 0.1g and subsequently euthanized by CO_2_ overdose. Following euthanasia, animals were decapitated and the heads were skinned and placed in 10% neutral buffered formalin with the jaws fully closed. Following fixation, the surface of the masseter was cleaned of any connective tissue and photographed in lateral view through a Leica MZ7.5 stereomicroscope using a SPOT Insight camera. The masseter, including superficial, anterior and posterior deep portions following Cox et al. [25], was dissected from the skull, blotted dry and weighed to the nearest 0.0001g.

**Fig 1:**
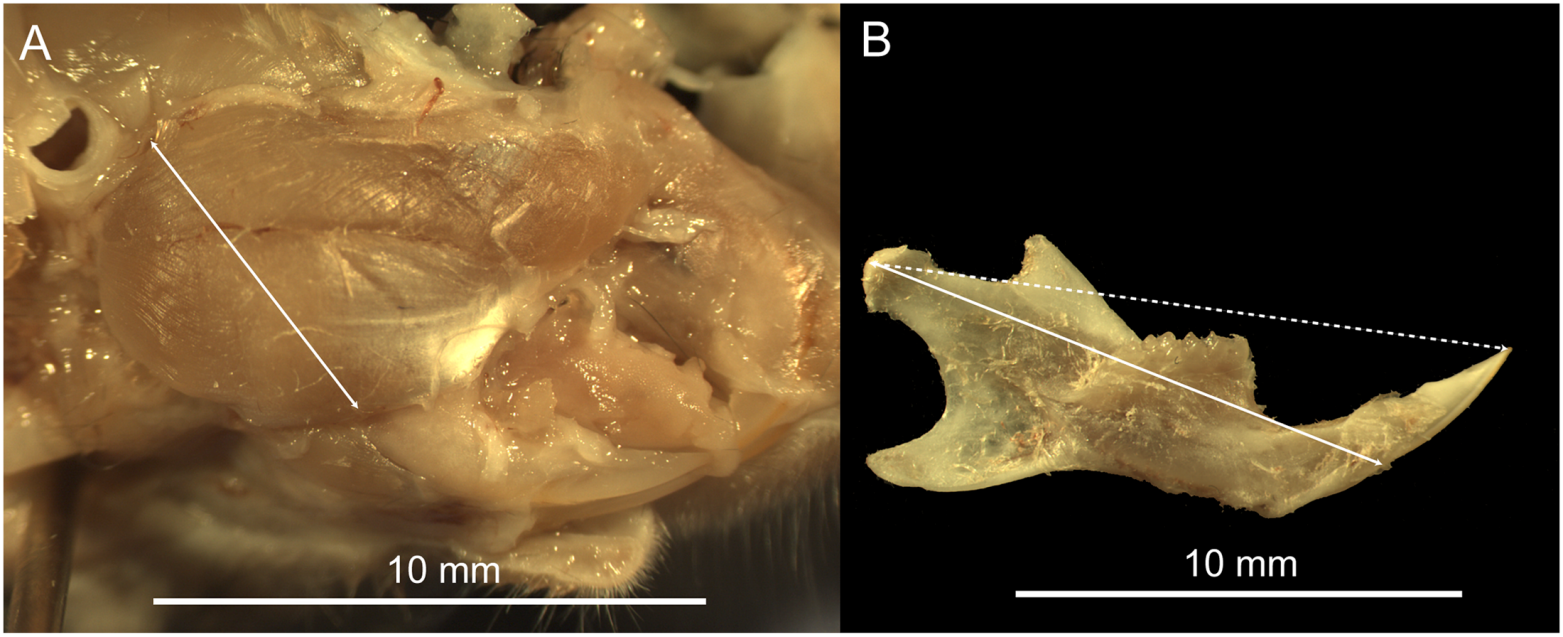
Measurements used to estimate the masseter moment arm (A) and load-arm length (B). The estimate for the masseter moment arm was measured from the jaw joint to the anteroinferior attachment of the superficial masseter on the jaw. The load-arm for incisor biting was measured in two ways as indicated by the solid and dashed lines. One measurement (dashed) included the incisor, whereas the other (solid) extended only to the inferior margin of the incisor alveolus.

Following masseter removal, the head was photographed through the microscope in lateral view. The jaw was then dissected free, cleaned of any soft tissue, allowed to dry, and photographed. Linear measurements were obtained from the digital images as shown in Fig 1. The masseter was measured from the condyle to the anteroinferior extent of masseter attachment on the jaw (Fig 1A). This served as a measure of the moment arm of the superficial masseter or in-lever [26]. Two estimates of the out-lever or the load arm for incisor biting were considered: 1) jaw length measured from the condyle to the inferior point on the incisor alveolus (condyle-alveolus) and 2) total jaw length from the condyle to the tip of the lower incisor taking into consideration incisor length and procumbency (condyle-incisor) (Fig 1B).

### Statistical Analysis

The independent variables body mass, absolute masseter mass, masseter mass normalized to body mass, absolute bite forces, bite force normalized to masseter mass, and food consumption were first tested for normality using the Shapiro-Wilk test. A Levene’s test was used to ensure that each combination of independent and dependent variable showed homogeneity of variances. With the exception of food consumption, all other variables were normally distributed (p>0.05) for all combinations of sex and treatment and showed homogeneity of variances (p>0.05). Therefore, we performed two-way ANOVAs on these independent variables to determine if there is an interaction between sex and treatment (control vs. DOX). Significant interactions were further investigated by evaluating the simple effects of treatment for male and female mice. Because food consumption data lacked both a normal distribution and homogeneity of variances, a non-parametric Kruskall-Wallis test was used to test for differences in feeding behaviors between DOX and control mice.

Given the association between Mstn deficiency and cranial skeletal morphology observed by Vecchione et al. [8], we also evaluated whether masticatory configuration differed between the control and treatment groups within each sex. For these analyses we compared the mechanical advantage associated with incisor bites as determined by the ratio of the moment arm of the masseter (in-lever) and the two estimates of the incisor bite load arm (out-lever). Analyses were conducted using an independent Student’s t test comparing control and DOX animals within each sex. We focused on within-sex comparisons for these analyses, rather than the sex by treatment interaction, because we were interested primarily in the biomechanical basis of potential differences in bite force. If there are no significant differences in skeletal measurements reflecting masticatory configuration between control and treatment groups, this would indicate that any differences in bite force are due to changes associated with the force production capabilities of the muscle.

As this study is exploratory in a novel conditional Mstn-deficiency model and given the conflicting results between cranial and post-cranial muscles for force production capabilities, we employ two-tailed tests of significance for all statistical analyses, with α = 0.05. All datasets analyzed in this study are provided in S1 File and S2 File.

## Results

**Fig 2:**
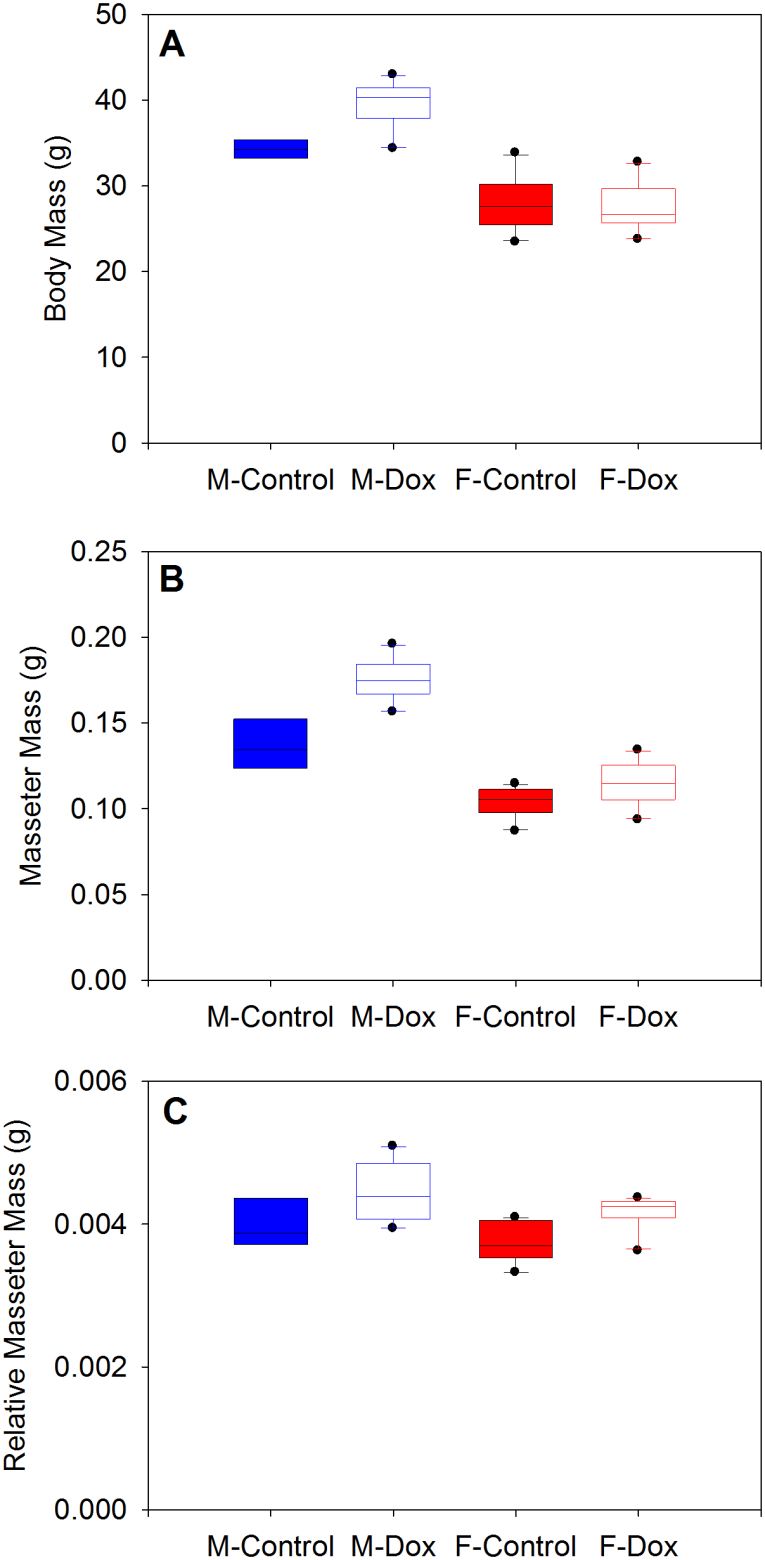
Body mass (A), masseter mass (B) and relative masseter mass (C) (i.e., masseter mass/body mass) in control and DOX mice. There was a significant sex x treatment interaction on body mass and absolute masseter mass. Body mass and masseter mass was significantly larger in DOX males compared to controls, but not in females. There was no interaction between sex and treatment on relative masseter mass. However, there was a significant main effect of sex and treatment.

There was a statistically significant interaction between sex and treatment on body mass (F_[1,34]_ = 10.757, p = 0.002). Analysis of the simple effects of treatment within each sex reveals that the DOX-treated males were significantly larger than controls (F_[1,34]_ = 17.642, p<0.001) but DOX and control females did not differ (F_[1,34]_ = 0.106, p = 0.746) (Fig 2A). There was also a significant interaction between sex and treatment on absolute masseter mass (F_[1,34]_ = 12.501, p = 0.001). DOX males had significantly larger masseters than control males (F_[1,34]_ = 45.181, p<0.001), but there was no difference within females (F_[1,34]_ = 3.902, p = 0.056) (Fig 2B). There was no interaction between sex and treatment on relative masseter mass (i.e., masseter mass/body mass) (F_[1,34]_ = 0.063, p = 0.803). However, there was a significant main effect of sex indicating that relative masseter mass is significantly larger in males than in females (F_[1,34]_ = 5.969, p = 0.020). Likewise, there was a significant main effect of treatment, with DOX-treated animals having larger relative masseter mass than controls (F_[1,34]_ = 18.610, p<0.001) (Fig 2C).

**Fig 3:**
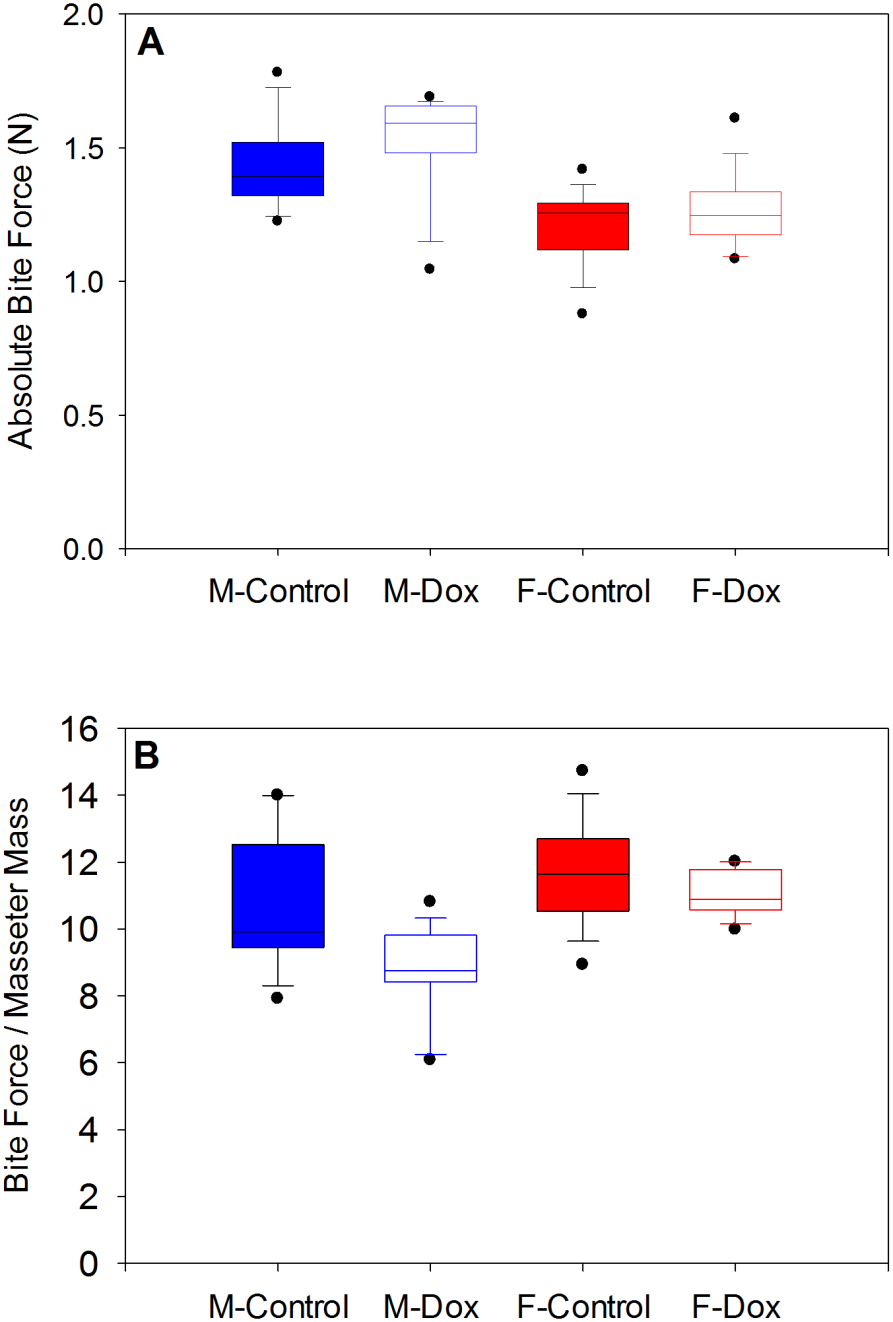
Absolute (A) and relative (B) bite forces in control and DOX mice. A two-way ANOVA revealed significant effects on absolute bite force for sex but not for treatment and no significant treatment x sex interaction. B. For relative bite forces, there was no treatment x sex interaction but significant main effects for both treatment and sex.

There was no interaction between sex and treatment on absolute bite force (F_[1,34]_ = 0.055, p = 0.815) nor was there a significant main effect of treatment (F_[1,34]_ = 1.373, p = 0.249). However, there was a significant main effect for sex (F_[1,34]_ = 17.612, p<0.001), with males having larger absolute bite forces than females (Fig 3A). There was also no interaction between sex and treatment on bite force normalized to masseter mass (F_[1,34]_ = 1.769, p = 0.192) but the main effects of treatment (F_[1,34]_ = 7.0602, p = 0.012) and sex (F_[1,34]_ = 10.563, p = 0.003) were significant. Relative bite force in female controls and female DOX animals were higher than in treatment-matched males. Within females and males, the controls had relatively higher bite forces than DOX-treated animals (see Fig 3B).

Food consumption in male mice was on average higher than in females within the control (male average: 35.39g ± 8.55; female average: 25.58g ± 3.73) and DOX (male average: 35.36g ± 7.66; female average: 26.58g ± 3.37) mice. The Kruskal-Wallis test revealed that the distribution of food consumption scores were not similar for all groups (control female, control male, DOX female, DOX male) (H[3] = 20.253, p<0.001). Bonferroni-adjusted post-hoc tests reveal significant differences between female and male controls (p = 0.016) and DOX-treated mice (p = 0.004) but not between control and DOX mice within each sex.

**Fig 4:**
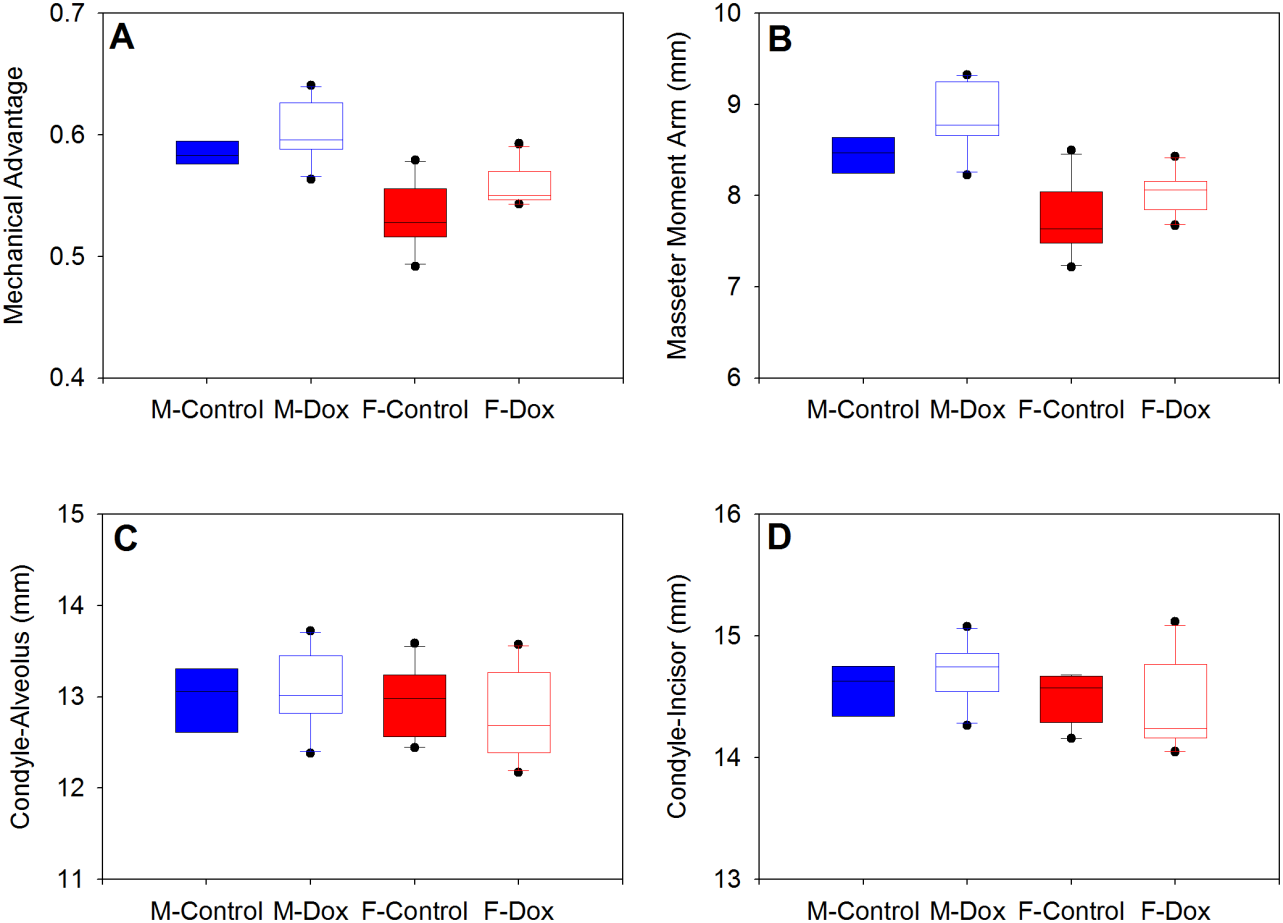
Masticatory configuration in control and DOX mice. Mechanical advantage (A) is significantly greater in DOX males and females due to significantly longer masseter moment arms (B). There were no differences between control and DOX mice in mandible length (C) or total jaw length (D) within each sex.

Within males and females, mechanical advantage was enhanced in the DOX-treated animals compared to controls (males: p = 0.047; females: p = 0.029; Fig 4A). This was due to significantly longer masseter moment arms (males: p = 0.013; females: p = 0.05; Fig 4B) and not to a decrease in the load arm associated with incisor biting (Fig 4C and 4D) as neither load-arm estimate differed significantly between DOX and control mice within males and females.

## Discussion

DOX-treatment had a sex-specific effect on body and masseter mass, with males showing more pronounced effects for both than females. Within males, the increase in body and masseter mass were on the order of 15% and 29%, respectively. Despite no change in overall body mass, DOX females show a slight tendency towards larger masseters than controls. Consequently, their relative masseter mass was significantly larger than controls and the differences between the two were on par with those observed in males. The increase in body mass in males but not in females, coupled with a smaller increase in masseter mass in females, suggests a potential role for sex hormone involvement in differential growth. Because these animals had been backcrossed to C57/BLB-J for 4 generations, we used JAX published growth curves as a comparison. On average C57/B6 male and female mice grow 2 grams from ages 4 to 5 months. The rate of growth at that stage is not higher in males than females, and thus does not account for the effects of mstn inhibition here reported. Thus, the relative impacts of testosterone versus estrogen need to be explored in the context of this Mstn inhibition protocol.

In contrast to bite forces in the constitutive *Mstn* ^-/-^ mouse (see [7]), we observed no change in absolute bite force after 12 weeks of Mstn inhibition. This was somewhat unexpected, especially for the males given their increased muscle mass. Nevertheless, it is consistent with some studies in *Mstn* ^-/-^ mice showing no increase in the absolute force-generating capacities of select post-cranial muscles (e.g., [4, 6, 27, 28]). When normalized to estimates of muscles size, controls had higher bite forces than DOX treated animals. Thus, an increase in muscle size was not accompanied by a proportionate increase in bite force. This was more pronounced in males than in females in large part due to their larger increase in muscle mass with DOX. These results are also consistent with the reports for *Mstn* ^-/-^ mice for force estimates from post-cranial muscles (e.g., [4, 5, 27, 28]) as well as for size-normalized temporalis-stimulated bite forces [7].

Because our experimental design relies on voluntary bite forces, it is prudent to assess whether we are capturing maximal, or at least comparable, biting performance across animals. To this end, we can directly compare our data to the stimulated bite forces from constitutive *Mstn* KO (see [7]). Maximum bite forces in our male control and DOX groups were similar to those recorded for the *Mstn*-/- males at the highest stimulation frequency (ranging from approximately 0.8 to 1.6 Newtons) and larger than the bite forces recorded at lower stimulation frequencies. Our females averaged slightly lower than the largest stimulated bite forces in the male *Mstn* ^-/-^ mice. Thus, given overall differences in size between males and females, it is likely that we have obtained reasonable representative maximal biting in our animals and that behavioral variability (e.g., willingness to bite) has not overly influenced our dataset. There is also no evidence of altered loading histories due to differences in the repetitiveness of chewing or gnawing between control and DOX mice. This would have been suggested by a significant difference in food consumption between the groups. To further examine the potential impact of altered loading histories between control and DOX-treated mice, future work will incorporate both hard and soft foods to the experimental design.

Comparison of DOX versus control mice revealed longer masseter moment arms in the DOX males and females due to a more anteriorly-placed masseter insertion. Given that there are no differences in either estimate of the load arm, the more anteriorly-placed masseter insertion in the DOX animals results in a net increase in the mechanical advantage for incisor biting. These results are interesting when viewed in light of the bite force data. All else being equal, increasing the mechanical advantage of the masseter should have substantially increased bite force in the DOX group. This suggests a number of things. First, the DOX group simply may not have bitten as hard as controls. Although we have no reason to believe this is the case based on the arguments outlined above, the effect of Mstn inhibition will be tested in more controlled experiments using stimulated bite forces complemented by whole muscle and single fiber studies to measure contractile properties. Second, the DOX treatment may have negatively impaired the contractile properties of the jaw muscles thereby limiting any impact of an increase in the masseter moment arm. If this is the case, then the jaw muscles may respond differently to postnatal Mstn inhibition compared to weight-bearing post-cranial muscles. Alternatively, DOX treatment may not induce a shift towards more type IIx/b fibers like that observed in the temporalis of constitutive Mstn^-/-^ mice, the latter which also show much more pronounced changes in muscle mass compared to the more modest increases observed here in the conditional KO (see [7, 11]). For example, in constitutive Mstn^-/-^ mice, the temporalis and masseter muscles are 40% and 80% larger, respectively, than WT individuals [7–9]. The extent to which shifts in myosin isoforms play a role remains to be seen, especially given that isoform transitions occur postnatally within the masseter, and that these changes differ from muscles in other regions of the body (e.g., limb muscles) [29, 30].

Finally, previous studies of constitutive *Mstn* ^-/-^ mice have inferred increased skull loading as a result of increased force production without alterations in the external stimulus. This is based on studies evaluating skeletal parameters in *Mstn* ^-/-^ and *Mstn* ^+/+^ mice, including cranial suture morphology, temporomandibular joint proportions, external skeletal dimension and skeletal biomineralization among other things [8, 9, 11–13]. Our bite force and food consumption data indicate that postnatal inhibition of Mstn may not result in increased loading simply due to increased muscle mass and thus we would predict few, if any, changes in the skull. This would not be surprising given that we only observed a 29% increase in masseter mass following DOX treatment in males (and 10% in females) compared to an almost 80% increase in *Mstn* ^-/-^ males [13]. If altered cranial loading histories do not occur with DOX treatment, this would be consistent with the results of Arounleut et al. [16] showing no differences in bone formation or resorption, bone strength, or skeletal weight following Mstn inhibition with a pharmacological Mstn inhibitor (propeptide-Fc) despite changes in skeletal muscle mass and fiber size.

Nevertheless, the small increases in absolute bite force and the significant increase in masseter mass in the DOX males suggest that additional work on postnatal Mstn inhibition on cranial biology is needed. The increase in masseter size alone indicates a positive impact on jaw muscle phenotype at the gross level. This complements recent research investigating the potential of postnatal Mstn inhibition in the treatment of sarcopenia showing overall positive influences on skeletal muscle (e.g., [16, 31, 32]). However, our data also suggest that the inclusion of a regimented over-loading or exercise-training experimental design may be required to directly counter muscle weakness. This could be accomplished by altering the dietary hardness or toughness of the food given to the animals, as they have been shown to sufficiently impact the cranial loading environment (e.g., [13, 33, 34–37]). Given the interest in neutralizing Mstn action as a therapeutic intervention for muscle wasting, further elucidating the consequences of its postnatal inhibition remains an important goal. Moreover, understanding the effects of postnatal Mstn inhibition coupled with rigorous loading and unloading regimens could prove to be particularly insightful for designing therapeutics for individuals dealing with age- or disease-related losses of muscle mass and/or strength.

## Supporting Information

S1 ARRIVE Checklist Completed “The ARRIVE Guidelines Checklist” for reporting animal data in this manuscript. (PDF)

S1 File Bite force and phenotypic data. Data provided are the average maximum bite force per animal, calculated as the average of the largest bite force recorded in 3 separate recording sessions for each animal. Phenotypic measurements included body mass, masseter mass, mechanical advantage for incisor biting, estimates of the masseter moment arm and two measures of jaw length.
(XLSX)

S2 File Food consumption data. Food consumption estimates were measured in grams for each cage of DOX and control mice. Food was weighed at initial provisioning and weekly thereafter before supplementing with a known amount. A weekly estimate of food consumed per animal was calculated as the total weight of food consumed each week divided by the number of animals in the cage.
(XLSX)

## References

[1] Grobet L, Poncelet D, Royo LJ, Brouwers B, Pirottin D, Michaux C, et al Molecular definition of an allelic series of mutations disrupting the myostatin function and causing double-muscling in cattle. Mamm Genome. 1998;9(3):210–3. 9501304 10.1007/s003359900727

[2] McPherron AC, Lawler AM, Lee SJ. Regulation of skeletal muscle mass in mice by a new TGF-beta superfamily member. Nature. 1997;387(6628):83–90. 9139826 10.1038/387083a0

[3] Baligand C, Gilson H, Menard JC, Schakman O, Wary C, Thissen JP, et al Functional assessment of skeletal muscle in intact mice lacking myostatin by concurrent NMR imaging and spectroscopy. Gene Ther. 2010;17(3):328–37. 10.1038/gt.2009.141 20010628

[4] Gentry BA, Ferreira JA, Phillips CL, Brown M. Hindlimb skeletal muscle function in myostatin-deficient mice. Muscle Nerve. 2011;43(1):49–57. 10.1002/mus.21796 21082689 PMC3052792

[5] Mendias CL, Marcin JE, Calerdon DR, Faulkner JA. Contractile properties of EDL and soleus muscles of myostatin-deficient mice. J Appl Physiol (1985). 2006;101(3):898–905. 16709649 10.1152/japplphysiol.00126.2006 PMC4088255

[6] Amthor H, Macharia R, Navarrete R, Schuelke M, Brown SC, Otto A, et al Lack of myostatin results in excessive muscle growth but impaired force generation. Proc Natl Acad Sci U S A. 2007;104(6):1835–40. 17267614 10.1073/pnas.0604893104 PMC1794294

[7] Byron CD, Hamrick MW, Wingard CJ. Alterations of temporalis muscle contractile force and histological content from the myostatin and Mdx deficient mouse. Arch Oral Biol. 2006;51(5):396–405. 16263075 10.1016/j.archoralbio.2005.09.006

[8] Vecchione L, Byron C, Cooper GM, Barbano T, Hamrick MW, Sciote JJ, et al Craniofacial morphology in myostatin-deficient mice. J Dent Res. 2007;86(11):1068–72. 17959898 10.1177/154405910708601109

[9] Vecchione L, Miller J, Byron C, Cooper GM, Barbano T, Cray J, et al Age-related changes in craniofacial morphology in GDF-8 (myostatin)-deficient mice. Anat Rec (Hoboken). 2010;293(1):32–41. 19899116 10.1002/ar.21024 PMC3113544

[10] Jeffery N, Mendias C. Endocranial and masticatory muscle volumes in myostatin-deficient mice. R Soc open sci. 2014;1:140187 10.1098/rsos.140187 26064569 PMC4448778

[11] Byron CD, Borke J, Yu J, Pashley D, Wingard CJ, Hamrick M. Effects of increased muscle mass on mouse sagittal suture morphology and mechanics. Anat Rec A Discov Mol Cell Evol Biol. 2004;279(1):676–84. 15224409 10.1002/ar.a.20055

[12] Byron CD, Maness H, Yu JC, Hamrick MW. Enlargement of the temporalis muscle and alterations in the lateral cranial vault. Integr Comp Biol. 2008;48(3):338–44. 10.1093/icb/icn020 21669796

[13] Ravosa MJ, Lopez EK, Menegaz RA, Stock SR, Stack MS, Hamrick MW. Using "Mighty Mouse" to understand masticatory plasticity: myostatin-deficient mice and musculoskeletal function. Integr Comp Biol. 2008;48(3):345–59. 10.1093/icb/icn050 21669797

[14] Jeffery NS, Stephenson RS, Gallagher JA, Jarvis JC, Cox PG. Micro-computed tomography with iodine staining resolves the arrangement of muscle fibres. J Biomech. 2011;44(1):189–92. 10.1016/j.jbiomech.2010.08.027 20846653

[15] Murakami M, Hirano H, Watanabe Y, Sakai K, Kim H, Katakura A. Relationship between chewing ability and sarcopenia in Japanese community-dwelling older adults. Geriatr Gerontol Int. 2014. 10.1111/ggi.12399 25363233

[16] Arounleut P, Bialek P, Liang LF, Upadhyay S, Fulzele S, Johnson M, et al A myostatin inhibitor (propeptide-Fc) increases muscle mass and muscle fiber size in aged mice but does not increase bone density or bone strength. Exp Gerontol. 2013;48(9):898–904. 10.1016/j.exger.2013.06.004 23832079 PMC3930487

[17] Personius KE, Jayaram A, Krull D, Brown R, Xu T, Han B, et al Grip force, EDL contractile properties, and voluntary wheel running after postdevelopmental myostatin depletion in mice. J Appl Physiol (1985). 2010;109(3):886–94. 20595537 10.1152/japplphysiol.00300.2010 PMC2944632

[18] Welle S, Bhatt K, Pinkert CA, Tawil R, Thornton CA. Muscle growth after postdevelopmental myostatin gene knockout. Am J Physiol Endocrinol Metab. 2007;292(4):E985–91. 17148752 10.1152/ajpendo.00531.2006

[19] Welle S, Burgess K, Thornton CA, Tawil R. Relation between extent of myostatin depletion and muscle growth in mature mice. Am J Physiol Endocrinol Metab. 2009;297(4):E935–40. 10.1152/ajpendo.00179.2009 19654287 PMC2763790

[20] Burgess K, Xu T, Brown R, Han B, Welle S. Effect of myostatin depletion on weight gain, hyperglycemia, and hepatic steatosis during five months of high-fat feeding in mice. PLoS One. 2011;6(2):e17090 10.1371/journal.pone.0017090 21390326 PMC3044753

[21] Hengstler JG, Van der Burg B, Steinberg P, Oesch F. Interspecies differences in cancer susceptibility and toxicity. Drug Metab Rev. 1999;31(4):917–70. 10575555 10.1081/dmr-100101946

[22] Huh WJ, Khurana SS, Geahlen JH, Kohli K, Waller RA, Mills JC. Tamoxifen induces rapid, reversible atrophy, and metaplasia in mouse stomach. Gastroenterology. 2012;142(1):21–4 e7 10.1053/j.gastro.2011.09.050 22001866 PMC3708546

[23] Dechow PC, Carlson DS. A method of bite force measurement in primates. J Biomech. 1983;16(10):797–802. 6643517 10.1016/0021-9290(83)90003-9

[24] Williams SH, Peiffer E, Ford S. Gape and bite force in the rodents *Onychomys leucogaster* and *Peromyscus maniculatus*: does jaw-muscle anatomy predict performance? J Morphol. 2009;270(11):1338–47. 10.1002/jmor.10761 19480012

[25] Cox PG, Jeffery N. Reviewing the Morphology of the Jaw-Closing Musculature in Squirrels, Rats, and Guinea Pigs with Contrast-Enhanced MicroCT. Anatomical Record-Advances in Integrative Anatomy and Evolutionary Biology. 2011;294(6):915–28. 10.1002/ar.21381 21538924

[26] Jablonski N. Evolution of the masticatory apparatus in Theropithecus In: Jablonski N, editor. Theropithecus: the rise and fall of a primate genus. New York: Cambridge University Press; 1993 p. 299–329.

[27] Matsakas A, Macharia R, Otto A, Elashry MI, Mouisel E, Romanello V, et al Exercise training attenuates the hypermuscular phenotype and restores skeletal muscle function in the myostatin null mouse. Exp Physiol. 2012;97(1):125–40. 10.1113/expphysiol.2011.063008 22058168

[28] Ploquin C, Chabi B, Fouret G, Vernus B, Feillet-Coudray C, Coudray C, et al Lack of myostatin alters intermyofibrillar mitochondria activity, unbalances redox status, and impairs tolerance to chronic repetitive contractions in muscle. Am J Physiol Endocrinol Metab. 2012;302(8):E1000–8. 10.1152/ajpendo.00652.2011 22318951

[29] Agbulut O, Noirez P, Beaumont F, Butler-Browne G. Myosin heavy chain isoforms in postnatal muscle development of mice. Biol Cell. 2003;95(6):399–406. 14519557 10.1016/s0248-4900(03)00087-x

[30] Miwa Y, Sunohara M, Sato I. Expression of myosin heavy chain isoforms in the postnatal mouse masseter muscle. Okajimas Folia Anat Jpn. 2009;86(3):105–10. 20166551 10.2535/ofaj.86.105

[31] Murphy KT, Chee A, Gleeson BG, Naim T, Swiderski K, Koopman R, et al Antibody-directed myostatin inhibition enhances muscle mass and function in tumor-bearing mice. Am J Physiol Regul Integr Comp Physiol. 2011;301(3):R716–26. 10.1152/ajpregu.00121.2011 21677277

[32] Schirwis E, Agbulut O, Vadrot N, Mouisel E, Hourde C, Bonnieu A, et al The beneficial effect of myostatin deficiency on maximal muscle force and power is attenuated with age. Exp Gerontol. 2013;48(2):183–90. 10.1016/j.exger.2012.11.008 23201547

[33] Bouvier M, Hylander WL. The effect of dietary consistency on morphology of the mandibular condylar cartilage in young macaques (*Macaca mulatta*). Prog Clin Biol Res. 1982;101:569–79. 7156160

[34] Bouvier M, Hylander WL. The effect of dietary consistency on gross and histologic morphology in the craniofacial region of young rats. Am J Anat. 1984;170:117–26. 6731337 10.1002/aja.1001700109

[35] Ciochon RL, Nisbett RA, Corruccini RS. Dietary consistency and craniofacial development related to masticatory function in minipigs. J Craniofac Genet Dev Biol. 1997;17(2):96–102. 9224944

[36] Tanaka E, Sano R, Kawai N, Langenbach GE, Brugman P, Tanne K, et al Effect of food consistency on the degree of mineralization in the rat mandible. Ann Biomed Eng. 2007;35(9):1617–21. 17522978 10.1007/s10439-007-9330-x

[37] Yamada K, Kimmel DB. The effect of dietary consistency on bone mass and turnover in the growing rat mandible. Arch Oral Biol. 1991;36(2):129–38. 1711839 10.1016/0003-9969(91)90075-6

